# Parallel and Divergent Evolutionary Solutions for the Optimization of an Engineered Central Metabolism in *Methylobacterium* *extorquens* AM1

**DOI:** 10.3390/microorganisms3020152

**Published:** 2015-04-09

**Authors:** Sean Michael Carroll, Lon M. Chubiz, Deepa Agashe, Christopher J. Marx

**Affiliations:** 1 Organismic and Evolutionary Biology, Harvard University, Cambridge, MA 02138, USA; E-Mails: scarroll@oeb.harvard.edu (S.M.C.); lchubiz.umsl@gmail.com (L.M.C.); dagashe@ncbs.res.in (D.A.); 2 Department of Biology, University of Missouri-St. Louis, St. Louis, MO 63103, USA; 3 National Centre for Biological Sciences, Bangalore 560065, India; 4 Faculty of Arts and Sciences Center for Systems Biology, Harvard University, Cambridge, MA 02138, USA; 5 Department of Biological Sciences, Institute for Bioinformatics and Evolutionary Studies, University of Idaho, Moscow, ID 83843, USA

**Keywords:** *Methylobacterium*, bioengineering, experimental evolution, genome sequencing, RNA polymerase, C_1_ metabolism

## Abstract

Bioengineering holds great promise to provide fast and efficient biocatalysts for methanol-based biotechnology, but necessitates proven methods to optimize physiology in engineered strains. Here, we highlight experimental evolution as an effective means for optimizing an engineered *Methylobacterium extorquens* AM1. Replacement of the native formaldehyde oxidation pathway with a functional analog substantially decreased growth in an engineered *Methylobacterium*, but growth rapidly recovered after six hundred generations of evolution on methanol. We used whole-genome sequencing to identify the basis of adaptation in eight replicate evolved strains, and examined genomic changes in light of other growth and physiological data. We observed great variety in the numbers and types of mutations that occurred, including instances of parallel mutations at targets that may have been “rationalized” by the bioengineer, plus other “illogical” mutations that demonstrate the ability of evolution to expose unforeseen optimization solutions. Notably, we investigated mutations to RNA polymerase, which provided a massive growth benefit but are linked to highly aberrant transcriptional profiles. Overall, we highlight the power of experimental evolution to present genetic and physiological solutions for strain optimization, particularly in systems where the challenges of engineering are too many or too difficult to overcome via traditional engineering methods.

## 1. Introduction

*Methylobacterium* strains have emerged as premier biocatalysts for methanol-based biotechnology. Tools for metabolic engineering [1,2,3,4,5,6], metabolic models [7,8,9], systems-level assays of physiology [5,10], whole-genome sequences [11,12], and a rich, 55 year history of research [13] have positioned this genus as a key model system for converting methanol into value-added products. As a facultative methylotroph, *Methylobacterium* strains are able to grow on both reduced one-carbon (C_1_) compounds like methanol, as well as multi-C compounds like succinate. Primarily using methanol as a feedstock, *Methylobacterium* has thus far been engineered to produce a variety of compounds [14], including polyhydroxybutyrate derivatives [15], amino acids [16], butanol [17], and various proteins [18,19]. Despite the many advantages of methanol and *Methylobacterium*, research in this area is still in its infancy, and further exemplars are needed to validate *Methylobacterium* as a flexible and practical solution for biotechnology.

The demands of bioengineering can place considerable stress on cells, and identifying the root cause of the underlying physiological stressors and inefficiencies can prove difficult [20]. Once these challenges are identified, they can be overcome using further engineering methods or directed evolution (*i.e*., artificial selection of artificially-generated mutants) at target loci to optimize the engineered physiology. However, when the challenges of bioengineering are difficult to identify, or too extensive to optimize through “rational” engineering, experimental evolution offers a productive route toward strain improvement. In most experimental evolution regimes, mutants are allowed to arise naturally in serially transferred cultures, and either increase or decrease in frequency in populations due to natural selection for increased growth rate [21,22]. In *M. extorquens* AM1, experimental evolution has been successfully employed to study fundamental questions in evolution [23,24,25,26], and to optimize strains engineered with alternate codon usage [27], or conferred with the ability to metabolize dichloromethane [28]. However, one avenue of research that would greatly facilitate the development of *Methylobacterium*-based biotechnology would be to establish the use of experimental evolution as an effective method for strain optimization: To directly optimize bacterial growth, to “diagnose” physiological stressors and inefficiencies from strain construction, and to present genetic and physiological mechanisms by which these challenges are overcome.

A model system has been developed to investigate the physiological consequences of metabolic engineering in a strain of *M.* *extorquens* AM1 with a major overhaul of C_1_ metabolism. In wild-type (WT) *M. extorquens* grown on methanol, methanol dehydrogenase produces formaldehyde as a highly toxic metabolic intermediate, which is quickly oxidized through a series of reactions to formate using the cofactor tetrahydromethanopterin (H_4_MPT) [29,30]. Formate can then be oxidized to CO_2_ for reducing power [31,32], or assimilated into biomass via tetrahydrofolate-mediated reactions and the serine cycle [7,33,34]. Construction of an engineered *Methylobacterium* (EM) strain involved eliminating the H_4_MPT pathway, then expressing a new pathway for formaldehyde oxidation in its place [25]. To accomplish this change, we deleted the first dedicated enzyme for the synthesis of H_4_MPT, and introduced a plasmid (pCM410) expressing two genes—*flhA*, encoding *S*-hydroxymethylglutathione dehydrogenase [35]; and *fghA*, encoding *S*-formylglutathione hydrolase [36]—from *Paracoccus denitrificans.* These enzymes co-opt glutathione (GSH), an endogenous cofactor in most bacteria, such that it now serves as the major formaldehyde carrier. By itself, the strain lacking H_4_MPT is unable to grow on methanol; however, the heterologous expression of *flhA* and *fghA* permitted growth on methanol via GSH, albeit three-fold more slowly [25]. Subsequent work sought to understand what, exactly, is limiting growth in the EM strain, and how it could be improved using experimental evolution in the laboratory.

Experimental evolution was carried out by inoculating 8 replicate flasks with EM and propagating these cultures via serial transfer on methanol for over 600 generations. Adaptation in these evolved “F” populations was both rapid and pronounced, resulting in strains that were up to 2.5-fold faster than EM by generation 600 (G600) [10]. Improvements were also most pronounced on methanol and methylamine *versus* other growth substrates, suggesting only minor optimization towards general growth conditions [10,25].

A number of studies have dissected aspects of the genetic and physiological basis of growth improvement in the evolved F strains, and these included a couple cases of highly parallel evolution. The most direct adaptation in the F strains was to lessen the burden of expressing the engineered pathway itself. While this pathway is essential in EM for growth on methanol, its initial expression level from the methanol dehydrogenase promoter was far too high. This led to selection for a menagerie of mutations that decreased expression in a variety of ways [37]. These rose in frequency in all F populations, and 8/8 of the strains examined here possessed one such change. The second example of a high degree of parallelism emerged upon discovering that the medium used for evolution was cobalt-limiting [38,39]. It was found that 6/8 F populations had IS insertions upstream of a novel transporter, *icuAB*, as had another 24/24 populations of WT evolved in separate experiments with methanol [39].

In addition to knowledge of parallel mutations at a couple loci, one study that fully sequenced the genome of an F4 isolate revealed a number of adaptations in host physiology [25]. Notable changes included mutations to *gshA* (encoding γ-glutamylcysteine ligase), which drives the second-to-last step in the synthesis of GSH; and to *pntAB* (encoding pyridine nucleotide transhydrogenase), that drives the proton-driven interconversion of NAD(H) and NADP(H); both mutations were found to be highly beneficial, and each increased the expression of these genes due to satisfy the cellular demands for GSH and NADPH, respectively.

To begin to connect evolved genotypes to phenotypes, DNA microarrays were used to examine both gene-by-gene and global patterns of transcriptional change from WT, to EM, to evolved isolates from each of the 8 F populations [10]. While the overarching trend for the F strains was to “restore” WT-like expression from a perturbed, EM transcriptional state, several large-scale differences in the evolved expression profiles were noted, causing strains F1, F4, and F8 to cluster away from other F strains in a principal component analysis. For F4, an extraordinary number of “novel” decreases in expression were found to stem from a loss of roughly 617 kilobases through a large-scale deletion [25,40]; upon the exclusion of these deleted genes, the expression profile of F4 was similar to that of other F isolates. For F1 and F8, however, we observed a large number of truly “novel” changes in gene expression relative to EM, and the cause for these unique transcriptional profiles was not immediately clear. For these and the remaining F lines, knowledge of the mutations that occurred could greatly aid our understanding of evolved physiologies.

Here, we expand whole-genome re-sequencing to reveal the genomic basis of host adaptation in the remaining F strains. In light of these sequences, we investigate broad-level variation in the number and types of mutations across F strains, targets that were mutated in parallel across lineages, loci that are of particular interest to *M. extorquens* physiology and C_1_ metabolism, and connections between genome sequences and global expression data from microarray analyses. Finally, we provide an in-depth analysis of mutations to RNA polymerase in strains F1 and F8, which appear to offer highly beneficial, yet massively pleiotropic changes in gene expression. Overall, our work draws attention to the usefulness of experimental evolution for *Methylobacterium*-based bioengineering: To optimize strain growth and physiology, to “diagnose” physiological stressors and inefficiencies from engineering, and as a means to discover novel connections between the *M. extorquens* genome, cellular physiology, and C_1_ metabolism.

## 2. Materials and Methods

### 2.1. Strain Construction and Evolution Regime

All strains and plasmids used in this study are listed in Table S1. All mutants were derived from a pink-pigmented, “wild-type” laboratory strain of *M. extorquens* AM1 (CM501), and a white-colored strain (CM502) with a neutral mutation in carotenoid biosynthesis [3]. From these WT strains, pink (CM701) and white (CM702) EM strains were designed by: (1) disabling the native H_4_MPT-based pathway of formaldehyde by deleting *mptG*, encoding β-ribofuranosylaminobenzene 5′-phosphate synthase, the first committed step in the synthesis of the H_4_MPT cofactor [41]; and (2) introducing plasmid pCM410 expressing the *flhA* (encoding *S*-hydroxymethyl-GSH dehdyrogenase) and *fghA* (encoding *S*-formyl-GSH hydrolase) genes from *Paracoccus denitrificans* [25].

Eight replicate “F” populations—F1 through F8—were founded from either the pink CM701 (odd) or white CM702 (even) EM ancestors, and propagated in 9.6 mL of Hypho medium in 50 mL Erlenmeyer flasks with 15 mM methanol at 30 °C and 225 rpm for over 600 generations [25]. Populations were streaked at various time points to check for contamination, and were frozen, along with isolates, to keep a “living fossil record” of the evolution experiment. An isolate from each evolved population at G600 was chosen for in-depth physiological analyses [10] and whole-genome sequencing (described below).

Other strains were generated as follows. Mutations from evolved F isolates were moved into their ancestral EM and other genetic backgrounds using pCM433, a tetracycline-based “suicide” vector for allelic exchange mediated by homologous recombination [3]. For each allelic exchange construct, a PCR product was designed to amplify the mutation plus roughly 500 bp upstream and downstream flanking regions from the evolved isolates in which they occurred. Amplicons were assembled into pCM433 using Gibson isothermal assembly [42], and introduced into the host *M. extorquens* strain using triparental conjugal matings with the helper plasmid pRK2073 [43]. After selection for single- and double-crossover events [3], single colonies were isolated and screened for successful incorporation of the mutation. For EM-based backgrounds, plasmid pCM410 was cured before allelic exchange, and then was subsequently returned using triparental matings.

### 2.2. Growth Conditions and Measurement of Specific Growth Rate

Growth was performed using a modified Hypho minimal medium described in [25]. Liquid cultures were grown using 10 mL Hypho in 50 mL Erlenmeyer flasks, plus the appropriate carbon substrate added just prior to growth: Either methanol (20 mM) or succinate (3.5 mM). When necessary, antibiotics were used at the following concentrations: Kanamycin, 50 μg/mL; tetracycline 12.5 μg/mL; streptomycin, 100 μg/mL.

All growth measurements were performed using inoculation, acclimation, and experimental growth phases. Briefly, all *M. extorquens* strains were inoculated from freezer stocks into flasks with 0.5× methanol and 0.5× succinate to allow for robust growth. Upon growth saturation, flasks were transferred to an acclimation phase growth with the carbon source to be tested (*i.e.*, methanol only) in either a flask or microtiter plate. Finally, acclimation cultures were transferred once more to the same conditions for experimental measurements. The increase in optical density of cultures (OD_600_) over time was monitored in 48-well microtiter plates using an automated, high-throughput growth system [44], and the specific growth rate calculated from the exponential growth phase [45]. Cellulase enzyme from *Aspergillus niger* (Sigma-Aldrich, St. Louis, MO, USA) was added to cultures at a concentration of 0.1 mg/mL to minimize cell clumping and increase the accuracy of OD_600_ measurements [46]. Data points are plotted with the mean plus SEM for at least triplicate growth measurements.

### 2.3. Stress Test with Hydrogen Peroxide

A stress test of strains against hydrogen peroxide was performed via a disk diffusion assay as in [47]. All strains were grown to saturation in flasks with methanol and mixed 1:20 into soft agar (0.75%) Hypho medium pre-warmed to 42 °C, and 4 mL of this cell suspension was evenly poured onto the surface of normal Hypho agar (1.6%) plates with 125 mM methanol, +/− kanamycin. Plates were allowed to solidify and dry, and then a small (5 mm), sterilized filter disc was added to directly to the center. To this filter disc we added 5 μL of 10 M hydrogen peroxide, and incubated plates at 30 °C; each strain was assayed in triplicate, and control plates received no hydrogen peroxide. After one week of growth, we measured the diameter (cm) of growth inhibition caused by hydrogen peroxide stress, and normalized values respective to each strain’s pink or white ancestor.

### 2.4. Whole-Genome Re-Sequencing

Whole-genome re-sequencing of F populations isolates was performed as follows with the exception of F4 (CM1145), which was sequenced previously [25]. Preparation of genomic DNA was performed using phenol-chloroform extraction with isopropanol precipitation [48]. Sequencing was performed out-of-house by GENEWIZ, Inc. (South Plainfield, NJ, USA) using Illumina HiSeq2000 to produce 50 bp single-end reads. All data has been deposited to the NCBI Sequencing Read Archive (PRJNA273781). Reads were mapped against an EM (CM701) reference genome using breseq v0.21 [49] with Bowtie 2 v2.0.0-beta7 [50]. Mutations called by breseq were individually assessed and those with marginal or conflicting quality (*i.e.*, equal or greater mix of WT with the mutant sequence reads) were excluded from downstream analyses. Other mutations that were shared among all or most F strains were likely acquired in the construction of EM from WT, and were also excluded [25]. Mutations to the foreign pathway and plasmid were identified previously [37], with the exception of a mutation in *flhA*. All mutations identified in this study are listed in Table S2.

### 2.5. Analysis of Microarray Data

Analysis of microarray data was performed as described previously [10] to determine global gene expression profiles in WT, EM, and isolates of each of the evolved (EVO) F strains. Briefly, differentially expressed genes were identified using moderated *t*-test of values in EM/WT and EM/EVO comparisons and assimilated across multiple probes per locus. These comparisons distinguish changes in gene expression arising from the immediate, physiological *acclimation* to the engineered pathway (EM/WT) from those arising from *evolutionary* changes in the F lineages (EM/EVO). By creating a “reaction norm” of differentially expressed genes between WT, EM, and each EVO strain, we identified 4 unique categories of gene expression change: (1) “novel” gene expression, in which expression was unaltered in acclimation (EM/WT) but significantly different in adaptation (EM/EVO); (2) “restored” patterns, in which expression was altered in EM/WT but returned to WT levels in the EVO strain; (3) “unrestored” patterns, in which expression was altered in EM/WT and remained unchanged in EVO; and (4) “reinforced” patterns, in which expression was altered in EM/WT and then was exacerbated in the same direction in the EM/EVO comparison.

## 3. Results and Discussion

### 3.1. Overview of Genomic Changes at 600 Generations

We used whole-genome re-sequencing to identify the genomic changes in isolates from seven of the F populations at G600 to examine general trends in F genome evolution. These data supplement the previous information on the F4 isolate [25], mutations in the introduced pathway/plasmid [37], and in a couple other loci that had been targeted [10]. First, we found that the number of mutations varied across strains: From 4 in F2, to 18 in F8 (Table 1), including mutations to the foreign plasmid and pathway [25,37]. The types of mutations ranged from SNPs, small insertions and deletions, duplications, and the movement of transposable insertion sequence (IS) elements, which in the case of F4 resulted in the deletion of some 617 kilobases through recombination between homologous ISs. In comparing the number of mutations in each strain to either the degree of growth improvement or novel gene expression, we found no correlation (Figure S1).

Several genomic targets were mutated highly in parallel across the evolved F isolates (Table 2). By far the most frequently mutated target was that of the engineered pathway and plasmid. As mentioned above, prior work identified that 8/8 populations mutated the introduced plasmid in the EM ancestor [37], and 6/8 F populations had an IS insertion at *icuAB* [25]. Through examining the microarray data, we found that the only two evolved isolates lacking an *icuAB* mutation—F5 and F6—did not increase the expression of this locus. We did, however, find in the whole-genome sequence of F5 an IS insertion between a manganese/divalent metal cation transporter (*mntH*) and its transcriptional regulator (*mntR*), which increases expression of the former (and decreases the latter) by roughly four-fold. This transporter was found to be active with Cobalt(II) in *Escherichia coli* [51], and might serve the same role as *icuAB* in this lineage.

**Figure 1:**
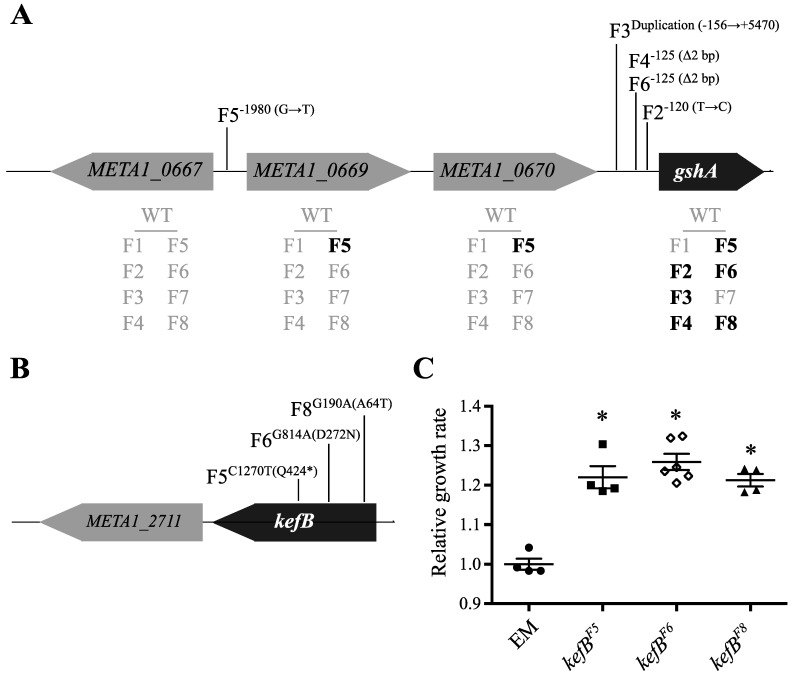
Parallel mutations to *gshA* and *kefB* occurred in multiple F lines. (**A**) Multiple mutations that appear to account for the observed increase in expression of *gshA*. Shown are evolved *gshA* alleles and increases in gene expression (bold) from microarray analysis. (**B**) The *kefB* locus was also targeted by parallel mutations in multiple F lines, and (**C**) each evolved allele was found to be beneficial in the EM background grown on methanol. Asterisk (*) indicates *p* < 0.05 in a two-tailed, unpaired *t*-test assuming unequal variance.

Another commonly mutated locus was *gshA*, encoding γ-glutamylcysteine ligase, which carries out the next-to-last step in GSH biosynthesis. Normally, GSH functions to protect the cell against oxidative stressors. In EM, however, GSH was also co-opted as a carrier of C_1_-units during formaldehyde oxidation, and is likely too dilute to efficiently carry out its native and engineered functions. Prior work identified a beneficial mutation in *gshA* in F4 [25], and here we identified four other mutations, including a gene duplication and a SNP roughly 2 kb upstream of *gshA*. All of these are associated with significantly increased (and “novel”) gene expression relative to EM as revealed by microarray analysis (Figure 1A) [10]. While mutations in the neighborhood of *gshA* appear in these 5/8 F strains, we also previously noted increased expression of *gshA* in F8 [10], suggesting that other mutations might affect the expression of this locus *in trans*. One remaining strain (F7) acquired an internal duplication to *cysE*—encoding serine acetyltransferase—which functions several reactions upstream of GshA in the synthesis of cysteine, and might serve the same functional role.

The potassium:proton antiporter, *kefB*, was found to be mutated in 3/8 F lineages. In *E. coli*, KefB activity is allosterically responsive to the GSH pool, whereby free GSH represses activity and oxidized conjugates (like CH_2_OH-GSH) are activators, causing cytoplasmic acidification and protection from oxidative stress [52,53]. In these evolved isolates (Figure 1B), it is unclear exactly how these missense and nonsense mutations alter KefB function; the F5 allele, in particular, prematurely ends the synthesis of KefB just before the GSH-binding regulatory domain. Each of these *kefB^EVO^* alleles was placed into the ancestral, EM background and provided an average 23% benefit during growth on methanol (Figure 1C). Similar mutations to *kefB* were identified in *M. extorquens* AM1 strains that were experimentally evolved to use methylamine as a carbon source via the *N*-methylglutamate pathway [54]. That allele was found to activate KefB activity and led to cytoplasmic acidification that counterbalanced the release of ammonia from methylamine.

**Table 1:** Summary of F population isolates at G600.

Population	Isolate	Growth Relative to EM ^1^	# Novel Expression ^1^	Total # Mutations	# Intergenic	# Coding	# IS Elements
F1	CM1727	1.96	149	11	2	8	2
F2	CM1730	2.15	12	4	2	1	1
F3	CM1139	2.44	17	11	5	5	2
F4	CM1145	2.50	217	9	4	4	2
F5	CM1739	2.09	42	11	5	5	6
F6	CM1742	2.09	47	11	2	8	3
F7	CM1745	2.16	21	9	2	6	2
F8	CM1748	2.11	197	18	8	8	6

^1^ Data from [10].

**Table 2:** Parallel evolution at specific genomic targets.

Isolate	pCM410	*icuAB*	*gshA*	META1_4902 ^1^	*kefB*	*rpoA*	ATP Synthase ^2^	*pntAB*	META2_0008 ^3^	META1_3102 ^4^
F1	+	+	−	+	−	+	+	−	−	−
F2	+	+	+	−	−	−	−	−	−	−
F3	+	+	+	+	−	−	+	+	+	−
F4	+	+	+	+	−	−	−	+	−	−
F5	+	−	+	−	+	−	−	−	+	−
F6	+	−	+	−	+	−	−	−	−	−
F7	+	+	−	−	−	−	−	−	−	+
F8	+	+	−	−	+	+	−	−	−	+

^1^ Putative membrane protein; ATPase; ^2^ Mutation to *atpI* in F1; *atpF* in F3; ^3^ Putative beta-ketoacyl synthase; ^4^ conserved hypothetical protein, putative cAMP-binding domain-like.

Finally, a number of other interesting loci were identified in only one or two evolved strains. As was noted previously, *pntAB* (encoding a pyridine nucleotide transhydrogenase) was mutated in 2/8 lineages to increase production of NADPH. Without H_4_MPT, neither of the methylene-H_4_MPT dehydrogenases can generate NADPH for the cell, making transhydrogenase essential for methylotrophy. Interestingly, we identified a 12 bp deletion within the coding frame of *mtdB* in strain F2, despite this locus having no known enzymatic role in the engineered formaldehyde oxidation pathway. It is not yet known whether this evolved *mtdB* allele was beneficial for optimizing EM physiology, or simply the result of relaxed selection due to disuse (*i.e.*, genetic drift). Other striking genomic changes included mutations to two different components of ATP synthase in F1 (*atpI*) and F3 (*atpF*); in F1, a mutation to glycine decarboxylase (*gcvP*) which, as part of the glycine cleavage system, was one of the largest changes in gene expression from WT to EM that remained unrestored in the evolved lineages [10]; mutations to lysine (F7) and leucine (F8) tRNAs; and finally, mutations to the alpha subunit of RNA polymerase in F1 and F8, which we explore in greater detail below.

### 3.2. Unique Expression Profiles Linked to Mutations in RNA Polymerase

**Figure 2:**
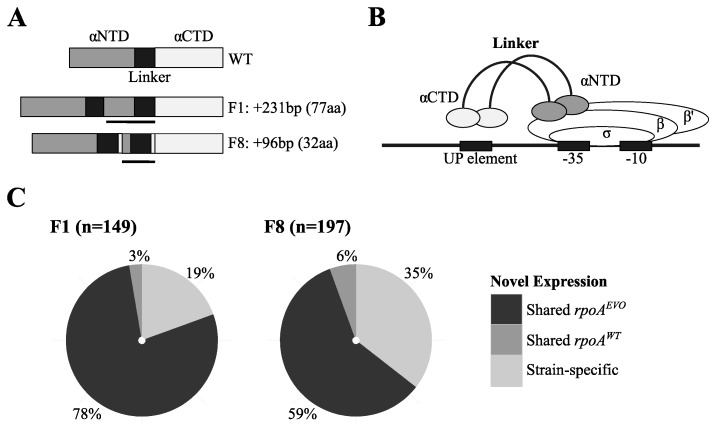
Mutations that extend the RNAP linker are linked to unique gene expression in F1 and F8. (**A**) Wild-type RpoA consists of αNTD and αCTD domains connected by an unstructured linker. F1 and F8 possess internal sequence duplications (black bar) that are hypothesized to extend both the linker and neighboring domains. (**B**) Bacterial RNAP is composed of five core subunits. Important to this work are the effects of linker extension mutations on the αNTD and the αCTD, which facilitate binding to DNA and co-regulatory proteins near UP binding sites. Modified from [58]. (**C**) F1 and F8 have up to 16-fold more genes with “novel” expression [10], most of which are uniquely shared between only these two strains relative to other F strains.

Prior work with DNA microarrays identified two evolved strains—F1 and F8—with highly divergent transcriptional profiles, however the cause for these unique transcriptional profiles was not immediately clear. Here, we identified a major candidate to explain this altered gene expression in mutations to the alpha subunit of RNAP, encoded by *rpoA*. Given its central role in DNA transcription, RNA polymerase (RNAP) has far-reaching control over global mRNA levels, and changes to this enzyme complex would be expected to have highly pleiotropic and generally deleterious effects. F1 and F8 acquired independent internal duplications in *rpoA* that extend the protein product by 77 (F1) or 32 (F8) amino acids (Figure 2A). Homology to more well-characterized RpoA proteins suggests that these mutations extend an unstructured linker region connecting two distinct RpoA domains: The *N*-terminal domain (αNTD) that helps to assemble the rest of the RNAP holozyme; and a C-terminal domain (αCTD) that binds to upstream promoter (UP) elements at target genes (Figure 2B). It is important to note, however, that extension of the linker is not perfect, and small portions of the neighboring domains are duplicated in both instances. While uncovering the exact biochemical effects of these mutations will require substantial future work, a broad hypothesis is that extensions of the linker domain change interactions between the αNTD of RNAP and gene-specific UP elements, altering gene expression at some loci, such as several A-rich regulatory sequences that were identified upstream of various C_1_-related genes [55]. Mutations to the *rpoA* linker have been shown to alter gene expression in other systems, and have varying effects across different types of promoter elements [56,57]. In light of these *rpoA* mutations, we revisited our previously generated microarray data for unique patterns of gene expression in strains F1 and F8.

The most unique feature of the transcriptomes of F1 and F8 was hundreds of instances of “novel” gene expression relative to other F strains. In light of newly found mutations to *rpoA*, we sought to reexamine these instances of novel expression in F1 and F8, to identify what proportion of these genes are both shared and novel in only these strains. Across genes with novel expression in F1 (*n* = 149) and F8 (*n* = 197), we found that the vast majority was shared between these and no other F strain (Figure 2C). These genes include a number of interesting functions, with most involved in C_1_ metabolism, DNA replication and repair, and the stress response (Table 3). A fairly large proportion of genes that were novel in only F1 or F8 could be due to mutations (or alleles) unique to each strain, differences in *rpoA^EVO^* alleles, or simply marginally (in)significant differences in gene expression identified in the microarray analysis. Finally, only a small handful of genes with novel expression were shared between these and other F strains. Given that many C_1_-related genes are uniquely differentially expressed only in these *rpoA^EVO^* strains, including genes like *mauF* (methylamine utilization)—which plays no known role in methanol oxidation—it is possible that *rpoA^EVO^* acts predominantly with higher-level regulators of cellular growth and C_1_-metabolism.

**Table 3:** Gene expression divergence in strains with *rpoA^EVO^* mutations.

Gene	Locus	Function	Expression Change ^1^	Exp F1 ^2^	Exp F8 ^2^	Exp Others ^2,3^
*recO*	META1_0821	DNA repair	Novel	−2.23	−2.14	−1.21
*dnaK*	META2_0894	Hsp70 chaperone protein	Novel	2.41	2.06	−1.02
*polA*	META2_0971	DNA polymerase	Novel	2.68	2.46	1.22
*META1_2781*	META1_2781	putative antioxidant enzyme; Tpx-related thiol peroxidase	Novel	5.17	4.06	1.33
*xoxF*	META1_1740	C_1_ metabolism/regulation	Novel	6.32	5.70	1.38
*mxbD*	META1_1753	C_1_ metabolism/regulation	Novel	−1.78	−1.96	1.00
*fdh4A*	META1_2094	C_1_ metabolism	Novel	−8.28	−8.28	−1.85
*mauF*	META1_2769	C_1_ metabolism	Novel	7.57	6.41	1.55
*gap*	META1_2218	PHB biosynthesis	Novel	−3.71	−3.94	−2.27
*META1_0222*	META1_0222	putative sensor histidine kinase	Unrestored	1.31	1.09	1.79
*def*	META1_1530	peptide deformylase	Restored	2.17	2.83	1.47
*ibpA*	META1_1514	heat shock protein	Restored	−2.53	−3.14	−1.61
*META1_4113*	META1_4113	hypothetical protein; putative sarcosine oxidase-related	Restored	−18.13	−10.34	−3.34
*META1_5150*	META1_5150	hypothetical protein	Restored	−35.75	−31.56	−2.69

^1^ As defined in “Materials and Methods” and [10]: “Novel” expression is significantly different in a comparison of EVO/EM but not EM/WT; “restored” expression is altered in EM/WT and returns to WT levels in EVO/EM; “unrestored” is altered in EM/WT and remains unchanged in EVO/EM; ^2^ Average fold change relative to EM. −, decrease in expression; ^3^ Excludes instances of F4 gene deletions.

Furthermore, we found that F1 and F8 uniquely restored WT gene expression. In contrast to strictly “novel” changes that arise solely from adaptation, we previously identified a set of 455 genes whose expression was perturbed during the physiological acclimation of EM from WT, and were either restored, unrestored, or reinforced through F evolution [10]. While all F lines generally restored perturbations in EM to WT expression, we found many instances where the magnitude of change differed in F1 and F8 relative to other F lines (Table 3). Notably, a number of genes were more strongly restored—*versus* only partially restored or unrestored—than other F strains. Our results suggest that, for a common set of gene expression changes that were caused by the genetic changes from WT to EM, F1 and F8 coped with these perturbations in slightly different, and perhaps more effective way.

### 3.3. Investigation of Conditions under which rpoA^EVO^ Was Advantageous

The presence of nearly identical *rpoA* mutations in two independently evolved populations strongly suggested that these genomic changes were beneficial for growth of EM on methanol. To confirm and quantify the potential benefit of these mutations, we used allelic exchange to transfer the alleles from F1 and F8 into the ancestral, EM background, and measured growth in these strains. However, as is often the case, the effect of beneficial mutations can be limited to particular genetic backgrounds or environments, especially for mutations to highly pleiotropic genes with far-reaching effects. To explore the universality of a growth benefit incurred by *rpoA^EVO^* we examined the effect of these mutations under a number of different genetic backgrounds and growth environments.

#### 3.3.1. Mutations in *rpoA* Were Highly Beneficial in EM Grown on Methanol

**Figure 3:**
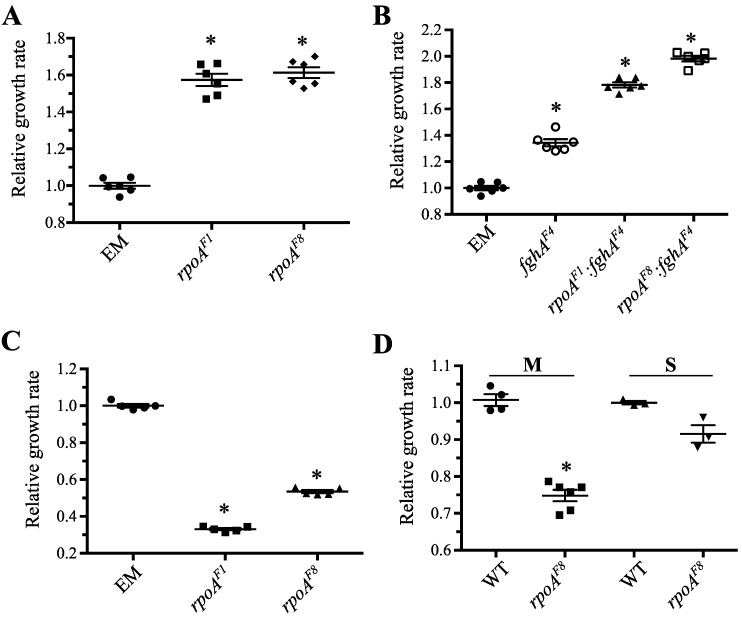
Effect of *rpoA^EVO^* across multiple genetic backgrounds and growth conditions. (**A**) Mutations to *rpoA* were highly beneficial in the ancestral, EM background. (**B**) *rpoA^EVO^* mutations remained beneficial in presence of beneficial (*fghA^F4^*) mutations to the engineered pathway. (**C**) *rpoA^EVO^* mutations were strongly deleterious in EM grown on succinate. (**D**) The *rpoA^F8^* allele was deleterious in the WT background grown on both methanol and succinate. Repeated attempts to generate a WT strain possessing *rpoA^F1^* failed. Asterisk (*) indicates *p* < 0.05 in a two-tailed, unpaired *t*-test assuming unequal variance.

Placed in the context of the ancestral, EM background, we found that both the *rpoA^F1^* and *rpoA^F8^* alleles substantially improve growth on methanol (Figure 3A). In fact, with respective increases of 57% and 61%, these F1 and F8 *rpoA* alleles are among the largest-effect beneficial mutations yet discovered in this system. Importantly, our measurements of specific growth rate on methanol suggest that these mutations are most important in the exponential phase of growth, and not necessarily for lag or stationary phase. Thus, faced with a serious growth defect and a multitude of physiological perturbations caused by the initial engineering of EM, evolved clones that acquired *rpoA^EVO^* would have experienced a significant growth advantage over their cohorts, despite the highly pleiotropic effect of these mutations. While further work is needed to understand the exact mechanism by which *rpoA^EVO^* mutations are beneficial, microarray analysis points to uniquely expressed genes in F1 and F8 with functions in C_1_ metabolism, cellular replication, and the stress response. These mutations could invoke in cells a response to “grow at all costs,” bypassing broad-level physiological mechanisms that direct the cell to either grow and replicate or preserve resources for increased stress resistance and survival [59].

#### 3.3.2. Mutations in *rpoA* Remained Advantageous in the Context of Other Beneficial Mutations to the Engineered Pathway

To begin to understand *why* such far-reaching, highly pleiotropic mutations are beneficial in this system, we sought to test the effect of *rpoA^EVO^* in the presence of other beneficial mutations with well-known physiological targets. To this end, we made mutational combinations with a well-characterized mutation from our system—*fghA^F4^*—that provides a growth benefit by decreasing the expression of the engineered formaldehyde oxidation pathway [25]. Combining mutations that both decreased protein over-expression costs have been found to be less beneficial than when they were present individually (*i.e.*, antagonistic epistasis) [25,60]. We reasoned that if the physiological effects of *rpoA^EVO^* were also focused to ameliorate the costs of expressing the foreign pathway, we should observe a diminished selective benefit when *rpoA^EVO^* was combined with *fghA^F4^* than when it was alone.

Our results suggested that the *rpoA^EVO^* and *fghA^F4^* mutations address largely different physiological stressors/selective pressures in the evolved populations. We found that the presence of *fghA^F4^* did not obviate the selective benefit of *rpoA^EVO^* (and *vice versa*), as both mutations remained highly beneficial in combination (Figure 3B). We did, however, observe some evidence of mutational interactions: The benefit of *rpoA^F1^* was reduced from 57% to 44% in the presence of *fghA^F4^*, indicative of “diminishing returns” epistasis among beneficial mutations [25,61]; whereas the benefit of *rpoA^F8^* was slightly increased from 61% to 64%. Overall, however, these results show that the benefit of *rpoA^EVO^* was not targeted specifically to optimize expression of the foreign pathway and plasmid.

#### 3.3.3. Mutations in *rpoA* Were Deleterious in EM Grown on Succinate

Given the transcriptional pleiotropy of the *rpoA^EVO^* alleles, we sought to understand whether these mutations remain advantageous on other growth substrates, particularly succinate. Metabolism of succinate is very different than that of methanol, as it bypasses C_1_ enzymes to feed directly into the TCA cycle [62,63], is under different regulatory control, and is thought to be limited by energy (ATP) instead of reducing equivalents (NAD(P)(H)) [64].

Consistent with the idea that pleiotropy is often deleterious in alternative environments, we found that *rpoA^EVO^* substantially hindered growth of EM on succinate. The F1 and F8 alleles reduced growth by 67% and 46%, respectively (Figure 3C). These data corroborated prior observations that F1 isolates are the worst on succinate, and actually worse than their EM ancestor, however F8 was not as nearly as bad [25]. These results suggest that the benefit of *rpoA^EVO^* mutations do not extend to succinate growth conditions, and could be specific to growth on methanol or C_1_ metabolism, in general.

#### 3.3.4. Mutations in *rpoA* Are Deleterious in WT Grown on Methanol and Succinate

To further assess the pleiotropic effects of *rpoA^EVO^*, we sought to determine whether these alleles remain beneficial in the context of the WT *M. extorquens* AM1 background. Despite numerous attempts, we were unable to isolate WT: *rpoA^F1^* clones through allelic exchange, and identified only a handful of successful WT: *rpoA^F8^* strains; far fewer than are normally found through this protocol. Upon analyzing the growth of the latter strain on methanol, we observed that *rpoA^F8^* is markedly deleterious (−25%) in the WT background (Figure 3D). This suggests that the underlying benefit of *rpoA^EVO^* is highly specific—advantageous only to the physiology of the EM background grown on methanol—and that the *rpoA^F1^* and *rpoA^F8^* mutations, despite their similarity, might invoke subtle yet important allele-specific effects.

#### 3.3.5. Mutations in *rpoA* Offer Protection against Hydrogen Peroxide Stress

A final test sought to determine whether *rpoA^EVO^* afforded cells increased protection against oxidative stressors. One such stressor is formaldehyde, which is a necessary, albeit toxic, intermediate in C_1_ metabolism that is rapidly produced during methylotrophic growth [65]. Any mechanisms that help to regulate flux through formaldehyde and limit its toxicity are likely perturbed in EM, as synthesis of the native carrier of formaldehyde (H_4_MPT) was abolished and the newly engineered carrier (GSH) was diluted from its normal function to protect against oxidative stress. Microarray analyses identified a number of genes involved in cell replication and the stress response that are differentially expressed in EM relative to WT, or in *rpoA^EVO^* relative to *rpoA^WT^* strains. Thus, we sought to test whether a general aspect of the benefit of *rpoA^EVO^* was by bolstering defenses against oxidative stress.

**Figure 4:**
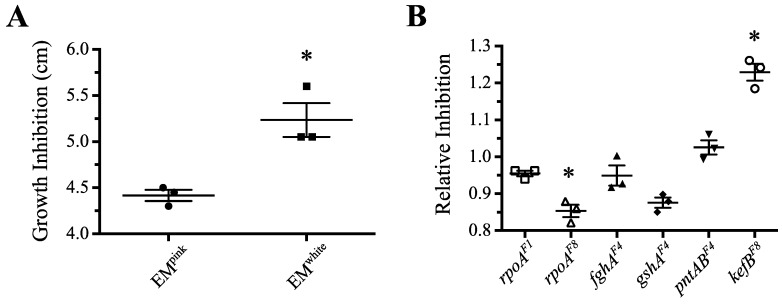
Stress test of *rpoA* and other mutants grown on methanol plates with hydrogen peroxide. (**A**) Interestingly, pink and white EM ancestors were not equivalent in these growth conditions, as the pink-pigmented strain with carotenoids was roughly 19% more resistant against hydrogen peroxide. (**B**) Upon normalizing the growth of mutants to their respective pink/white ancestor, several mutants—including *rpoA^EVO^*—offered protection against hydrogen peroxide. Asterisk (*) indicates *p* < 0.05 in a two-tailed, unpaired *t* test assuming unequal variance. Note that the *gshA^F4^* allele imparted a marginally insignificant reduction in inhibition (*p* = 0.056).

We tested resistance to hydrogen peroxide (H_2_O_2_) using a disc assay in which a lawn of cells suspended in soft agar were spread onto methanol agar plates with a small filter disc containing 5 μL of 10 M hydrogen peroxide placed in the middle [47]. In control plates with no peroxide, all strains produced a lawn of growth over the entire plate; however, in experimental plates, we observed a well-defined zone of growth inhibition from H_2_O_2_-induced growth inhibition. The diameter of this “dead zone” was measured and averaged over triplicate plates, and expressed relative to each strain’s respective pink or white EM ancestor. One initial observation was that the pink and white EM ancestors—which were used to prevent cross-contamination in the evolution experiments and were shown to be neutral under standard growth conditions [25]—were, in fact, not equivalent under peroxide duress. In fact, the carotenoids present in the pink-pigmented EM offered roughly 19% more protection against peroxide than the otherwise isogenic white strain (Figure 4A). Moving forward, each beneficial mutation was tested in the pink or white ancestor in which it arose, and normalized relative to their specific ancestor.

Interestingly, we found that both *rpoA^EVO^* alleles, as well as a number of other mutations, offered a protective effect against H_2_O_2_ stress. The evolved *rpoA* alleles from F1 and F8 offered roughly 5% and 15% more protection against peroxide than their respective pink and white EM ancestors (Figure 4B). A number of other mutations, proven to be advantageous for growth, were also beneficial on hydrogen peroxide, including mutations to the foreign pathway (*fghA*) and glutathione biosynthesis (*gshA*). However, another mutation, *kefB^F8^*, was deleterious during H_2_O_2_ stress, reducing the growth zone by 23%. These results suggest that *rpoA^EVO^* may in part be advantageous for growth by helping to mount a physiological response to oxidative stressors. Analysis of other beneficial mutations suggests that increasing production of the GSH (*gshA*), or general improvements to decrease the metabolic burden of bioengineering (*fghA*) can also offer increased resistance to H_2_O_2_, whereas mutations to *pntAB* and *kefB*—both of which could alter the oxidative state of the cell via the flux of protons across the cell membrane—are slightly to strongly deleterious under conditions of H_2_O_2_ stress.

### 3.4. Complex Evolutionary Dynamics of rpoA^EV^° Mutations

Given that *rpoA^EVO^* mutations rose to an appreciable frequency (if not fixation) in F1 and F8 sampled at G600, we wondered whether similar mutations may have transiently appeared at earlier time points in other F populations. We were particularly curious about a prior study examining allele frequencies and clonal interference in population F4, in which a large number of mutants (14/72) screened at G150 had none of the known, beneficial mutations that were present in the majority of the population at G600 [66]. Using primers to amplify the linker region of *rpoA*, we found that each of these 14 mutants shared a single novel, *rpoA^F4^* allele that, like those in F1 and F8, extended the RpoA linker domain by 141 bp, or 47 amino acids.

**Figure 5:**
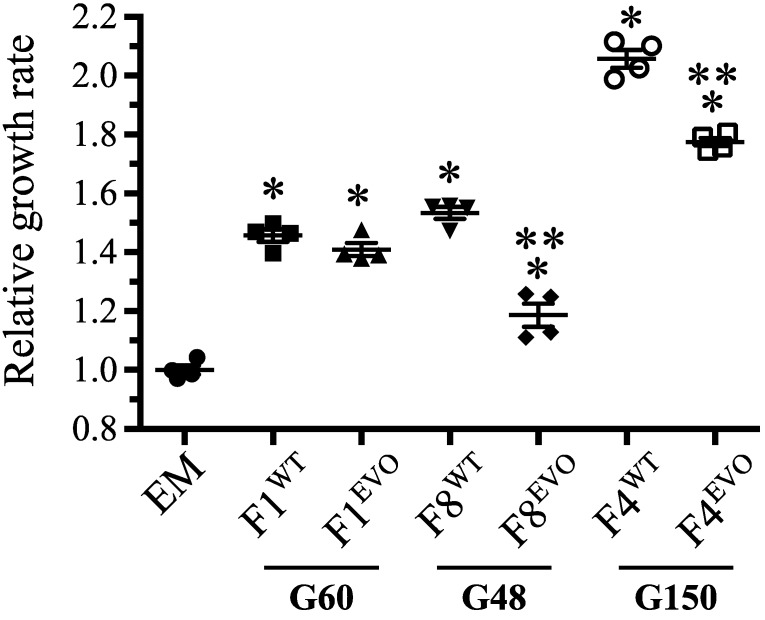
Growth of *rpoA^EVO^ and rpoA^WT^* cohorts sampled from early time points of populations F1, F4, and F8. Contemporaneous evolved isolates possessing either *rpoA^EVO^* or *rpoA^WT^* alleles were isolated from early F populations and assayed for their growth improvements relative to EM on methanol. In all populations, evolved isolates with the *rpoA^WT^* clones held a growth advantage over those with *rpoA^EVO^* present at the same time point. Single and double asterisks indicate *p* < 0.05 in a two-tailed, unpaired *t* test assuming unequal variance in comparing against EM (*) and early isolate cohorts (**), respectively.

To better understand the evolutionary dynamics of these alleles, we compared the growth of evolved clones possessing either *rpoA^EVO^* or *rpoA^WT^* in early populations of F1, F4, and F8. Prior work in *E. coli* has shown that co-occurring lineages can have differential evolvability, such that a clone from the “eventual winner” lineage was repeatedly able to adapt faster than a clone from the same time point that was part of the “eventual loser” lineage [67]. In our populations, we know that lineages containing *rpoA^EVO^* alleles were eventual winners in the F1 and F8 populations, but that the *rpoA^EVO^* from F4 was an eventual loser. Using samples archived as a “living fossil record” for the F populations, we were able to identify *rpoA^F1^* clones at very early evolutionary time points, and chose representative clones from each population with either *rpoA^EVO^* or *rpoA^WT^* to compare the performance of these cohorts relative to EM, and to one another. Although the earliest evolved clones substantially outperformed EM, the eventual fate of the lineages with or without *rpoA^EVO^* alleles was far from clear at that point. Strains from F1 at G60 had very similar fitness regardless of the *rpoA* allele present, whereas for the other two populations the isolate with an *rpoA^WT^* allele was faster than the one with *rpoA^EVO^*, despite the fact that in one population (F8) the *rpoA^EVO^* lineage overcame this disadvantage, and in the other (F4) they were outcompeted by a lineage with an *rpoA^WT^* allele (Figure 5). This suggests that *rpoA^EVO^* mutations arose in at least three F populations: Becoming fixed (or nearly so) in F1 and F8, while rising to a fairly high frequency in F4 before losing out to other clonal lineages. Given these complex dynamics, it is possible that still other *rpoA^EVO^* alleles experienced transient success in the remaining F populations before going extinct, as has been seen for other loci [23].

## 4. Conclusions

The study of replicate, independently evolved populations of *M. extorquens* allowed us to identify parallel evolutionary solutions in adapting to an engineered C_1_ metabolism. Repeatability of evolution in the F isolates highlights common genetic solutions to relieve physiological stressors and inefficiencies arising from the construction of EM, or from the evolution regime itself (*i.e.*, cobalt limitation). Interestingly, unlike other experiments where there were suites of mutations that occurred together more commonly than by chance [68], thereby suggesting alternative mutational paths to improvement, we did not observe any obvious patterns of this sort with our comparatively small number of replicate populations. Furthermore, parallelism does not necessarily imply equivalency of mutations. Take, for example, the four unique mutations that targeted the *gshA* locus: which varied not only in the strength by which they increase gene expression, from 2.5-fold in F2 to 15.1-fold in F6; but also in the scope of loci showing increased expression, from several loci upstream in F5, to several loci downstream in F3. While each of these alleles are sufficient to improve growth and physiology in the eyes of natural selection, the bioengineer has the added fortune of applying *post hoc* analyses to determine the optimal strength and scope of a mutational effect. The combined effects of a diversity of targets and variation in the effect of evolved alleles may collectively explain the prior observation that there was a far greater variance in fitness across these populations than commonly observed in experimental evolution [23].

In the case of *rpoA*, we show that the scope of a mutation can sometimes reach to genome-wide effects. Selected mutations that extended the linker domain of RpoA offered a massive growth benefit in EM while helping to protect against oxidative stressors; however, given the central role of RNAP in all of DNA transcription, they are likely to have far-reaching and possibly deleterious side effects. Interestingly, major perturbations such as those from metabolic engineering or acute environmental stress may place a premium on highly pleiotropic mutations that alter many aspects of physiology all at once. Various evolution experiments in *E. coli* have yielded a number of similarly pleiotropic mutations targeting components of RNAP, DNA replication, and other centrally-important genes [68,69,70,71]. In addition to far-reaching physiological effects, highly pleiotropic mutations might also have long-term consequences for evolution by creating selection for compensatory mutations, and by perhaps limiting the rate at which further adaptations can accrue [67]. Whether or not these limitations apply to bioengineering has yet to be shown. Yet, for all the potential drawbacks of highly pleiotropic mutations to organismal physiology and long-term evolution, a mechanistic investigation of *why* these mutations are beneficial can lead to more targeted solutions in strain optimization.

Overall, our work illustrates the benefits of using experimental evolution as a tool for biological engineering. As long as the desired outcomes of metabolic engineering are tied to growth, natural selection will quickly and efficiently enrich for mutants that are better able to grow and divide using the engineered physiology. While these evolved populations and isolates might in themselves be of use, the bioengineer is able to use whole-genome re-sequencing and assays of physiology to survey the products of adaptation and identify and selectively combine those traits and alleles that are most desirable. Mechanistic analyses of laboratory evolution can highlight the importance of genes that were known *a priori*, as well as reveal novel genes and hidden physiological connections that might not have been found through traditional engineering practices. By directly working to optimize the growth and physiology of strains, “diagnosing” physiological stressors and inefficiencies, and offering diverse solutions to these physiological challenges, experimental evolution is an invaluable tool for bioengineering and the continued development of *Methylobacterium*-based biotechnology.

## Supplementary Files

Supplementary File 1
