# Supplementary material for: Parallel and Divergent Evolutionary Solutions for the Optimization of an Engineered Central Metabolism in Methylobacterium extorquens AM1
Source: Microorganisms. 2015 Apr 9;3(2):152–74. doi: 10.3390/microorganisms3020152 (PMC5023240; doi:10.3390/microorganisms3020152)
Supplement: Supplementary File 1 [file microorganisms-03-00152-s001.docx]

**Supplementary Information**

**Table S1.** Strains and plasmids relevant to this study.

| **Strain** | **Description** | **Reference** |
| --- | --- | --- |
| CM501 | Pink, wild-type *M. extorquens* AM1 | [1] |
| CM502 | White version of CM501; *crtI*^502^ | [1] |
| CM508 | CM501 Δ*mptG* | [1] |
| CM624 | CM501 *crtI*^502^ Δ*mptG* | [2] |
| CM701 | CM508 pCM410; pink EM | [2] |
| CM702 | CM624 pCM410; white EM strain | [2] |
| CM1139 | Evolved isolate from F3 population at gen. 600 | [3] |
| CM1145 | Evolved isolate from F4 population at gen. 600 | [2] |
| CM1275 | CM702 *fghA^F4^* | [2] |
| CM1312 | CM702 *pntAB^F4^* | [2] |
| CM1316 | CM702 *gshA^F4^* | [2] |
| CM1727 | Evolved isolate from F1 population at gen. 600 | [3] |
| CM1730 | Evolved isolate from F2 population at gen. 600 | [3] |
| CM1739 | Evolved isolate from F5 population at gen. 600 | [3] |
| CM1742 | Evolved isolate from F6 population at gen. 600 | [3] |
| CM1745 | Evolved isolate from F7 population at gen. 600 | [3] |
| CM1748 | Evolved isolate from F8 population at gen. 600 | [3] |
| CM3305 | Evolved isolate from F4 population at gen. 150 with *rpoA^WT^* | [4] |
| CM3317 | Evolved isolate from F4 population at gen. 150 with *rpoA^EVO^* | [4] |
| CM3947 | CM702 *kefB^F8^* | This study |
| CM3990 | CM701 *kefB^F5^* | This study |
| CM4071 | CM702 *kefB^F6^* | This study |
| CM4073 | Evolved isolate from F1 population at gen. 60 with *rpoA^WT^* | This study |
| CM4077 | Evolved isolate from F1 population at gen. 60 with *rpoA^EVO^* | This study |
| CM4081 | Evolved isolate from F8 population at gen. 48 with *rpoA^WT^* | This study |
| CM4085 | Evolved isolate from F8 population at gen. 48 with *rpoA^EVO^* | This study |
| CM4091 | CM501 *rpoA^F8^* | This study |
| CM4099 | CM701 *rpoA^F1^* | This study |
| CM4101 | CM701 *rpoA^F1^ fghA^F4^* | This study |
| CM4164 | CM702 *rpoA^F8^* | This study |
| CM4166 | CM702 *rpoA^F8^ fghA^F4^* | This study |
| pCM433 | Vector for allelic exchange | [1] |
| pRK2073 | Helper plasmid for triparental matings | [5] |
| pCM410 | Expression plasmid for engineered formaldehyde oxidation pathway | [2] |
| pSC26 | pCM433 with *kefB^F5^* | This study |
| pSC29 | pCM433 with *kefB^F6^* | This study |
| pOB3 | pCM433 with *kefB^F8^* | This study |
| pSC51 | pCM433 with *rpoA^F1^* | This study |
| pSC52 | pCM433 with *rpoA^F8^* | This study |
| Reference numbers refer to main text. | | |

**Table S2**. Mutations in isolates of F1-F8. Excludes previously identified pCM410 mutations. Mutations from F4 identified in [25].

| **Strain** | **Chromosome** | **Position** | **Mutation** | **Details** | **Gene/Locus** | **Description** |
| --- | --- | --- | --- | --- | --- | --- |
| F1 | META1 | 618,949 | Δ3 bp | coding (1024–1026/1677 nt) | *META1_0585* | putative methyl‑accepting chemotaxis receptor/sensory transducer |
| F1 | META1 | 657,256 | +G | coding (139/2847 nt) | *gcvP* | glycine decarboxylase, PLP‑dependent, subunit (protein P) of glycine cleavage complex |
| F1 | META1 | 1122227 | Insertion ISMex4 | intergenic 4bp | *ureA/META1_1076* | urease gamma subunit/conserved hypothetical exported protein, putative outer membrane receptor protein |
| F1 | META1 | 2249143 | RpoA internal duplication | coding 230bp | *rpoA* | DNA-directed RNA polymerase alpha chain (RNAP alpha subunit) (transcriptase alpha chain) (RNA polymerase alpha subunit) |
| F1 | META1 | 2,635,578 | G→A | G130G (GGC→GGT) | *META1_2502* | putative capsule polysaccharide export outer membrane protein |
| F1 | META1 | 3,523,300 | C→T | G85S (GGC→AGC) | *atpI/META1_3389* | putative FoF1 ATP synthase, subunit I (*atpI*) |
| F1 | META1 | 4,756,847 | C→T | intergenic (−647/−16) | *META1_4632/hemB* | putative enoyl‑CoA hydratase/isomerase family protein/porphobilinogen synthase |
| F1 | META1 | 5,020,247 | Δ1 bp | coding (2321/2604 nt) | *META1_4902* | conserved hypothetical protein; putative membrane protein; putative ATPase |
| F1 | META2 | 305475 | Insertion ISMex3 | coding 10bp | *phnC* | phosphonate/organophosphate ester transporter subunit ; ATP‑binding component of ABC superfamily |
| F1 | META2 | 663,664 | G→A | G46S (GGC→AGC) | *META2_0706* | conserved hypothetical protein |
| F2 | META1 | 701,484 | T→C | intergenic (+67/−120) | *META1_0670/gshA* | putative l‑asparaginase (AnsA‑like)/gamma‑glutamylcysteine synthetase |
| F2 | META1 | 1122227 | Insertion ISMex4 | intergenic 4bp | *ureA/META1_1076* | urease gamma subunit/conserved hypothetical exported protein, putative outer membrane receptor protein |
| F2 | META1 | 1,834,813 | Δ12 bp | coding (419–430/894 nt) | *mtdB* | NAD(P)‑dependent methylene tetrahydromethanopterin dehydrogenase |
| F3 | META1 | 701448 | Duplication GshA | both 6996bp | *META1_0670/gshA: cobV* | putative l‑asparaginase (AnsA‑like)/gamma‑glutamylcysteine synthetase: Cobalamin synthase |
| F3 | META1 | 1122227 | Insertion ISMex4 | intergenic 4bp | *ureA/META1_1076* | urease gamma subunit/conserved hypothetical exported protein, putative outer membrane receptor protein |
| F3 | META1 | 3,087,651 | A→G | intergenic (+51/−61) | *META1_2955/pntAA* | hypothetical protein, putative membrane protein/NAD(P) + transhydrogenase, subunit alpha part 1. |
| F3 | META1 | 3,348,283 | C→T | T25T (ACC→ACT) | *acyP* | acylphosphatase (acylphosphate phosphohydrolase) |
| F3 | META1 | 3,521,307 | G→A | T172T (ACC→ACT) | *atpF/META1_3386* | putative FoF1 ATP synthase, subunit b (*atpF*) |
| F3 | META1 | 5,020,829 | G→T | A580E (GCG→GAG) | *META1_4902* | conserved hypothetical protein; putative membrane protein; putative ATPase |
| F3 | META1 | 5,312,062 | C→T | intergenic (−16/+160) | *META1_5154/META1_5155* | conserved hypothetical protein/hypothetical protein |

**Table S2.** *Cont.*

| **Strain** | **Chromosome** | **Position** | **Mutation** | **Details** | **Gene/Locus** | **Description** |
| --- | --- | --- | --- | --- | --- | --- |
| F3 | META2 | 11433 | Insertion ISMex16; deletion 2.015 kb | coding 2016bp | *META2_0008* | putative beta‑ketoacyl synthase |
| F3 | META2 | 174,546 | T→C | intergenic (+536/+540) | *META2_0192/META2_0193* | hypothetical protein/transposase of ISMex31, IS110 family |
| F3 | META2 | 725,292 | T→C | T7A (ACC→GCC) | *META2_0764* | hypothetical protein |
| F4 | META1 | 701,477 | Δ2 bp | intergenic | *META1_0670/gshA* | putative l‑asparaginase (AnsA‑like)/gamma‑glutamylcysteine synthetase |
| F4 | META1 | 1122226 | Insertion ISMex4 | intergenic | *ureA/META1_1076* | urease gamma subunit/conserved hypothetical exported protein, putative outer membrane receptor protein |
| F4 | META1 | 2860154 | Insertion ISMex2 | intergenic | *ccmC/ccmD* | putative role in type 1 cytochrome *c* biogenesis; heme transport |
| F4 | META1 | 3,087,664 | C→T | intergenic | *pntAA* | hypothetical protein, putative membrane protein/NAD(P) + transhydrogenase, subunit alpha part 1. |
| F4 | META1 | 5022190 | +6 bp | coding/insertion | *META1_4902* | conserved hypothetical protein; putative membrane protein; putative ATPase |
| F4 | META2 | 372,544 | C→T | C→T | *META2_0422* | hypothetical protein |
| F4 | META2 | GIANT DELETION |  | 617 kbbp | *Many* | varied |
| F4 | p3META |  | loss of p3META plasmid | 25 kbbp | *Many* | varied |
| F5 | META1 | 699,624 | G→T | intergenic (−376/−28) | *META1_0667/META1_0669* | hypothetical protein precursor; putative exported protein/conserved hypothetical protein |
| F5 | META1 | 1692714 | Insertion ISMex1 | coding 3bp | *META1_1617* | hypothetical protein |
| F5 | META1 | 1988578 | Insertion ISMex1 | pseudogene 3bp | *META1_1914* | fragment of conserved protein of unknown function (N‑terminal fragment) |
| F5 | META1 | 2,837,321 | G→A | Q424→Stop (CAG→TAG) | *kefB* | potassium:proton antiporter |
| F5 | META1 | 3966023 | Insertion ISMex14 | intergenic 7bp | *mntR/mntH* | DNA‑binding transcriptional regulator of mntH/manganese transport protein mntH |
| F5 | META1 | 4155174 | Insertion ISMex1; IS mess | intergenic 3bp | *META1_4075/META1_4076* | hypothetical protein/fragment of transposase of ISMex5, IS3 family (ORF 2) |
| F5 | META1 | 4156105 | Insertion ISMex1 | coding 3bp | *META1_4077* | hypothetical protein |
| F5 | META1 | 4,837,987 | Δ18 bp | coding (1164–1181/1221 nt) | *META1_4713* | conserved hypothetical protein |
| F5 | META1 | 5317679 | Insertion ISMex36/ISMex4 | coding 5bp | *pstS* | phosphate ABC transporter, periplasmic phosphate binding protein |
| F5 | META2 | 6,647 | A→G | intergenic (+1113/−1825) | *META2_0006/META2_0008* | hypothetical protein/putative beta‑ketoacyl synthase |
| F6 | META1 | 701,476 | Δ2 bp | intergenic (+59/−127) | *META1_0670/gshA* | putative l‑asparaginase (AnsA‑like)/gamma‑glutamylcysteine synthetase |
| F6 | META1 | 844,275 | T→C | E179G (GAG→GGG) | *META1_0810* | putative transcriptional regulator, Fur family; zinc uptake regulator ZUR |

**Table S2.** *Cont.*

| **Strain** | **Chromosome** | **Position** | **Mutation** | **Details** | **Gene/Locus** | **Description** |
| --- | --- | --- | --- | --- | --- | --- |
| F6 | META1 | 2,121,469 | T→C | V216A (GTG→GCG) | *ppk* | polyphosphate kinase, component of RNA degradosome |
| F6 | META1 | 2,837,777 | C→T | D272N (GAC→AAC) | *kefB* | potassium:proton antiporter |
| F6 | META1 | 3,844,019 | Δ12 bp | coding (746‑757/897 nt) | *META1_3695* | hypothetical protein; putative exported protein |
| F6 | META1 | 4110991 | Insertion ISMex1 | coding 3bp | *META1_4017* | hypothetical protein |
| F6 | META1 | 4,125,608 | T→C | intergenic (−3514/+420) | *META1_4038/rffH* | fragment of transposase related to IS701 family/glucose‑1‑phosphate thymidylyltransferase |
| F6 | META2 | 258184 | Insertion ISMex5; deletion 2.358 kb | coding 2359bp | *META2_0288/META2_0289* | putative general secretion protein D/General secretion pathway protein G |
| F6 | META2 | 422808 | Insertion ISMex1 | coding 3bp | *META2_0469* | conserved hypothetical protein |
| F6 | META2 | 802,994 | C→G | G198G (GGG→GGC) | *META2_0841* | putative metallophosphoesterase |
| F7 | META1 | 861,156 | G→C | G91R (GGC→CGC) | *META1_0828* | conserved hypothetical protein; putative exported protein |
| F7 | META1 | 1122227 | Insertion ISMex4 | intergenic 4bp | *ureA/META1_1076* | urease gamma subunit/conserved hypothetical exported protein, putative outer membrane receptor protein |
| F7 | META1 | 2,186,941 | 21 bp × 2 | duplication | *cysE* | serine acetyltransferase |
| F7 | META1 | 3,242,983 | C→T | R93H (CGC→CAC) | *META1_3102* | conserved hypothetical protein, putative cAMP-binding domain-like |
| F7 | META1 | 3,420,937 | G→A | intergenic (+130/−910) | *META1_tRNA16/META1_3288* | Lys tRNA/conserved hypothetical protein |
| F7 | META1 | 5,031,468 | A→C | I300S (ATC→AGC) | *META1_4910* | transposase of ISMex2, IS481 family |
| F7 | META1 | 5,141,378 | G→T | G144V (GGA→GTA) | *META1_5007* | hypothetical protein |
| F7 | META2 | 1136637 | Insertion ISMex1 | coding 3bp | *META2_1223* | conserved hypothetical protein |
| F8 | META1 | 165,584 | Δ1 bp | intergenic (−110/−2452) | *META1_0161/META1_0162* | phage integrase family protein/putative resolvase |
| F8 | META1 | 542,279 | C→T | D110D (GAC→GAT) | *META1_0519* | conserved hypothetical protein |
| F8 | META1 | 573,238 | T→C | noncoding (38/85 nt) | *META1_tRNA50* | Leu tRNA |
| F8 | META1 | 1122227 | Insertion ISMex4 | intergenic 4bp | *ureA/META1_1076* | urease gamma subunit/conserved hypothetical exported protein, putative outer membrane receptor protein |
| F8 | META1 | 1139697 | Insertion ISMex4 | intergenic 3bp | *META1_1088/META1_1089* | transposase of ISMex4, IS1380 family/conserved hypothetical protein |
| F8 | META1 | 1,305,011 | Δ10 bp | coding (150–159/528 nt) | *META1_1255* | hypothetical protein |

**Table S2.** *Cont.*

| **Strain** | **Chromosome** | **Position** | **Mutation** | **Details** | **Gene/Locus** | **Description** |
| --- | --- | --- | --- | --- | --- | --- |
| F8 | META1 | 1,976,919 | T→G | intergenic (−134/+477) | *META1_1899/META1_1901* | transposase of ISMex2, IS481 family/fragment of conserved hypothetical protein (C-terminal fragment) |
| F8 | META1 | 2249066 | RpoA internal duplication | coding 116bp | *rpoA* | DNA-directed RNA polymerase alpha chain (RNAP alpha subunit) (transcriptase alpha chain) (RNA polymerase alpha subunit) |
| F8 | META1 | 2,385,984 | C→T | intergenic (+423/+10) | *META1_2315/META1_2316* | putative catalase precursor/conserved hypothetical protein |
| F8 | META1 | 2,838,401 | C→T | A64T (GCC→ACC) | *kefB* | potassium:proton antiporter |
| F8 | META1 | 3243312 | Insertion ISMex3 | intergenic 9bp | *META1_3102/META1_3103* | conserved hypothetical protein, putative cAMP-binding domain-like/protein of unknown function |
| F8 | META1 | 4,062,362 | G→A | P123L (CCG→CTG) | *META1_3944* | hypothetical protein |
| F8 | META2 | 101564 | Insertion ISMex4 | coding 3bp | *META2_0097* | putative outer membrane receptor for iron transport |
| F8 | META2 | 649230 | Insertion of ISMex16; deletion 5.445 kb | IS/deletion 5446bp | *META2_0689-98* | hypothetical protein/hypothetical protein |
| F8 | META2 | 826095 | Insertion ISMex1 | coding 3bp | *META2_0868* | twin‑arginine translocation pathway signal precursor (fragment) |
| F8 | META2 | 1,071,173 | C→T | intergenic (−61/+80) | *META2_1142/META2_1143* | hypothetical protein/hypothetical protein |
| F8 | pCM410 | 3588 | G→A | A323T (GCC→ACC) | *flhA* | *S*-hydroxymethylglutathione dehydrogenase from *Paracoccus denitrificans* |
| **Excluded mutations—Mix of wildtype and mutant reads** | | | |  |  |  |
| F2 | META1 | 2949139 | giant duplication(?) | 267800bp | *ancA* | aconitate hydratase |
| F5 | META2 | 75303 | Insertion ISMex16; possible inversion | 4038bp | *META2_0064-69* | signal receiver domain protein/hypothetical protein; hypothetical protein |

**Figure S1**. Plot of the total number of mutations identified versus growth improvement
(**A**) and the number of loci with novel expression (**B**). Shown are the lines of best fit plus *r*^2^ and p values calculated from linear regression and Pearson correlation. *Y*-values are from [3] and listed in Table 1.

References

1. Marx, C.J. Development of a broad-host-range *sacB*-based vector for unmarked allelic exchange. *BMC Res. Notes* **2008**, *1*, 1.
2. Chou, H.-H.; Chiu, H.-C.; Delaney, N.F.; Segrè, D.; Marx, C.J. Diminishing returns epistasis among beneficial mutations decelerates adaptation. *Science* **2011**, *332*, 1190–1192.
3. Carroll, S.M.; Marx, C.J. Evolution after introduction of a novel metabolic pathway consistently leads to restoration of wild-type physiology. *PLoS Genet.* **2013**, *9*, e1003427.
4. Chubiz, L.M.; Lee, M.-C.; Delaney, N.F.; Marx, C.J. FREQ-Seq: A rapid, cost-effective,
   sequencing-based method to determine allele frequencies directly from mixed populations. **2012**, *7*, e47959.
5. Figurski, D.H.; Helinski, D.R. Replication of an origin-containing derivative of plasmid RK2 dependent on a plasmid function provided in *trans*. *Proc. Natl. Acad. Sci. USA* **1979**, *76*,
   1648–1652.

© 2015 by the authors; licensee MDPI, Basel, Switzerland. This article is an open access article distributed under the terms and conditions of the Creative Commons Attribution license (http://creativecommons.org/licenses/by/4.0/).
